# Multimodal non-invasive assessment of intracranial hypertension: an observational study

**DOI:** 10.1186/s13054-020-03105-z

**Published:** 2020-06-26

**Authors:** Chiara Robba, Selene Pozzebon, Bedrana Moro, Jean-Louis Vincent, Jacques Creteur, Fabio Silvio Taccone

**Affiliations:** 1 Policlinico San Martino, IRCCS For Oncology and Neuroscience, Department of Integrated Surgical and Diagnostic Science, University of Genova, Genova, Italy; 2 Neurosciences Critical Care Unit, Addenbrooke’s Hospital, University of Cambridge, Cambridge, UK; 3 Department of Intensive Care Medicine, Erasme Hospital, Université Libre de Bruxelles, Route de Lennik, 808, 1070 Brussels, Belgium

**Keywords:** Neuro-ICU, Non-invasive intracranial pressure, Brain injury, Pupillometer, Optic nerve sheath diameter

## Abstract

**Background:**

Although placement of an intra-cerebral catheter remains the gold standard method for measuring intracranial pressure (ICP), several non-invasive techniques can provide useful estimates. The aim of this study was to compare the accuracy of four non-invasive methods to assess intracranial hypertension.

**Methods:**

We reviewed prospectively collected data on adult intensive care unit (ICU) patients with traumatic brain injury (TBI), subarachnoid hemorrhage (SAH), or intracerebral hemorrhage (ICH) in whom invasive ICP monitoring had been initiated and estimates had been simultaneously collected from the following non-invasive indices: optic nerve sheath diameter (ONSD), pulsatility index (PI), estimated ICP (eICP) using transcranial Doppler, and the neurological pupil index (NPI) measured using automated pupillometry. Intracranial hypertension was defined as an invasively measured ICP > 20 mmHg.

**Results:**

We studied 100 patients (TBI = 30; SAH = 47; ICH = 23) with a median age of 52 years. The median invasively measured ICP was 17 [12–25] mmHg and intracranial hypertension was present in 37 patients. Median values from the non-invasive techniques were ONSD 5.2 [4.8–5.8] mm, PI 1.1 [0.9–1.4], eICP 21 [14–29] mmHg, and NPI 4.2 [3.8–4.6]. There was a significant correlation between all the non-invasive techniques and invasive ICP (ONSD, *r* = 0.54; PI, *r* = 0.50; eICP, *r* = 0.61; NPI, *r* = − 0.41—*p* < 0.001 for all). The area under the curve (AUC) to estimate intracranial hypertension was 0.78 [CIs = 0.68–0.88] for ONSD, 0.85 [95% CIs 0.77–0.93] for PI, 0.86 [95% CIs 0.77–0.93] for eICP, and 0.71 [95% CIs 0.60–0.82] for NPI. When the various techniques were combined, the highest AUC (0.91 [0.84–0.97]) was obtained with the combination of ONSD with eICP.

**Conclusions:**

Non-invasive techniques are correlated with ICP and have an acceptable accuracy to estimate intracranial hypertension. The multimodal combination of ONSD and eICP may increase the accuracy to estimate the occurrence of intracranial hypertension.

## Introduction

Intracranial hypertension is a common and severe complication after acute brain injury [1, 2]. Although there is no definitive evidence supporting the usefulness of ICP monitoring to improve patient outcomes [3], ICP measurement is considered as standard of care to guide therapy in patients with severe brain injury [4].

At present, the gold standard for ICP assessment is the placement of invasive devices, including external ventricular drains, which also enable drainage of the cerebrospinal fluid, or intraparenchymal micro-transducers [4]. The lack of clear indications on ICP monitoring in the latest guidelines [4] contributes to marked differences in practices related to the insertion of invasive ICP monitoring. Also, placement of these devices may take some time, consume resources, and carry certain risks, including local bleeding and/or infections [5–8].

A non-invasive, accurate, and safe tool to assess ICP would therefore be useful in this context. Various methods have been proposed, with different advantages and limitations, although none of them has been shown to be sufficiently accurate to replace invasive ICP measurement [9–13]. Transcranial Doppler (TCD)-derived indices, such as pulsatility index (PI) or a formula based on the diastolic flow velocity, and measurement of the optic nerve sheath diameter (ONSD), either by ultrasound, computed tomography (CT), or magnetic resonance imaging (MRI), have been shown to be reliable in the estimation of intracranial hypertension [11, 14, 15], as have alterations in pupillary reactivity and constriction velocity detected by automated pupillometers [16, 17]. However, these techniques have often been studied only in one specific condition (i.e., traumatic brain injury [TBI]) and rarely compared in the same cohort of patients.

The aim of this study was therefore to compare the accuracy of different non-invasive methods to estimate ICP and to estimate the occurrence of intracranial hypertension in a heterogeneous cohort of brain-injured patients. The following non-invasive methods were used: (a) ONSD measurement using ultrasound, (b) PI and the estimated ICP (eICP) [11] using TCD, and (c) the neurological pupil index (NPI) using automated pupillometry.

## Methods

This study was performed according to the STROBE reporting guidelines for observational studies.

### Study population

All consecutive adult (> 18 years) patients admitted to the Department of Intensive Care of the Hôpital Erasme (Université Libre de Bruxelles) over a period of 20 months (January 2017 to September 2018) were eligible if (a) they underwent invasive ICP monitoring for risk of intracranial hypertension; (b) they had a TBI, subarachnoid hemorrhage (SAH), or intracerebral hemorrhage (ICH); and (c) they had a “stable ICP” value (see below). Patients with other reasons for ICP monitoring, with known pupillary abnormalities (such as Adie’s pupil, Argyll Robertson pupil, post-surgical deformation, glaucoma), multiple sclerosis, ocular surgery, or severe peri-orbital edema limiting pupillary assessment were excluded. Patients without a temporal window for TCD assessment and without an arterial catheter for continuous blood pressure measurement were also excluded.

The decision to insert invasive ICP monitoring was made by the attending ICU physician in collaboration with the neurosurgeons. The decision was based on the latest version of the Brain Trauma Foundation guidelines [4] for TBI patients, while for SAH and ICH, this mainly based on patient’s clinical status on arrival (i.e., alteration of consciousness or rapid neuro-worsening) and neuroradiological findings (i.e., midline shift, impelling herniation, hydrocephalus). ICP was measured using an intraparenchymal fiber-optic transducer (Neurovent, Raumedic SA, Switzerland), or an extra-ventricular drainage (EVD) catheter inserted into the brain ventricles and connected to an external pressure transducer and drainage system. For non-invasive ICP assessment, patients needed to have a stable ICP value (< 10% variation) for at least 30 min and to not require specific ICP-driven therapies, suctioning, or other physical interventions. Concomitant therapies were not modified during the measurements.

Within the first 72 h after ICP insertion, the same experienced operator (FST) performed TCD, ONSD, and automated pupillometry (i.e., one measurement per patient) to help to better understand brain hemodynamics and compliance during ICP monitoring. In some patients with uncontrolled intracranial hypertension, the same tests were performed to evaluate the compromise of cerebral hemodynamics. These tests are all routinely used to monitor brain-injured patients in the ICU. The measurements were recorded bilaterally as, although an asymmetry between the right and left side of each variable may exist, ICP is considered as the result of the transmission of the pressure in the whole cranial cavity and the average of bilateral measurements could mirror more precisely brain pathophysiology, as previously suggested [10]. These data were collected when ICP did not vary by more than 3 mmHg during the measurements (around 8 min).

Invasive ICP data were collected simultaneously and, if variability exceeded this value, the non-invasive results were excluded. Intracranial hypertension was defined as ICP > 20 mmHg. EVD was closed during the measurement for correct ICP measurement. Systemic hemodynamic monitoring consisted of invasive arterial blood pressure from the radial artery, continuous electrocardiography, end-tidal CO_2_ monitoring, and pulse oximetry (SpO_2_). The local Ethics Committee approved the study (P2019/308), but waived informed consent.

### Pupillometry

Quantitative automated pupillary light reactivity was measured in both eyes using the NeurOptics NPi-200 (Neuroptics, Irvine, CA, USA) pupillometer, which uses a calibrated light stimulation of fixed intensity (1000 Lux) and duration (3.2 s) enabling rapid and precise measurement (0.05 mm limit) of pupil size, quantitative pupillary light reflex (PLR), constriction velocity, and latency [18]. Using an integrated algorithm, this pupillometer device also provides the NPI, which integrates all pupil variables (baseline size, constriction percentage, latency, constriction velocity, and dilation velocity) and varies from a value of 0 to 5 (with 0.1 decimal precision); an NPI score < 3 indicates abnormal pupillary function whereas NPI scores ≥ 3 are considered within the normal range. NPI value was average from measurements on both eyes.

### Optic nerve sheath diameter

ONSD was assessed using a 7.5-MHz linear ultrasound probe (Philips iE33, Paris, France) and the lowest acoustic power able to measure the ONSD. With the patient in the supine position and with head elevated to 30°, the probe was oriented perpendicularly in the vertical plane and at around 30° in the horizontal plane on the closed eyelids of both eyes without exerting pressure. Measurements were made in the axial and sagittal planes on the ultrasound images, 3 mm behind the retina in both eyes using an electronic caliper, and the final ONSD value was calculated by averaging four measured values as previously described [10]. Abnormal ONSD was considered if ≥ 6.0 mm.

### Transcranial Doppler

TCD was performed by the same investigator using the temporal window on both sides and an echo-color Doppler device with a 2-MHz transducer (Philips iE33, Paris, France). The TCD measurements were performed bilaterally on the middle cerebral artery (MCA); the mean flow velocity in the MCA was recorded 1 cm after the internal carotid artery bifurcation, and PI was automatically calculated using the formula: PI = [(systolic velocity − diastolic velocity)/mean velocity]. The final flow velocities were calculated by averaging the two measured values. PI was considered abnormal if > 1.2. eICP and estimated cerebral perfusion pressure (eCPP) were calculated using a validated formula [11], which includes simultaneous TCD and mean arterial pressure (MAP) readings to standardize measurements. Abnormal eICP was defined as > 20 mmHg.

### Data collection

All data were collected in an ICU patient data monitoring system (PDMS, Picis Critical Care Manager, Picis Inc., Wakefield, USA). Demographics, main comorbidities, and clinical characteristics on admission and on the day of ICP assessment were collected. Use of drugs that might interfere with pupillary constriction (i.e., opioids, sedatives, or barbiturates) and of neuromuscular blocking agents (NMBAs) was also noted. The initial severity of the brain injury was assessed using the Marshall score [19], the Fisher score [20], and the location and volume of the intracranial hemorrhage [21] for TBI, SAH, and ICH, respectively. We also recorded the use of mechanical ventilation, any osmotic therapy (mannitol or hypertonic saline), and of hypothermia (body temperature < 35 °C) during the ICU stay. Mortality was assessed at ICU discharge. Neurological outcome was assessed at hospital discharge using the Glasgow Outcome Scale (GOS).

### Statistical analysis

Data are expressed as mean ± standard deviation, median [interquartile range] or count (percentage), as appropriate. For continuous variables, normality assumption checking was performed by inspection of residual and normal plots. Differences between groups (TBI, SAH, and ICH) were assessed using a Fisher’s exact test for categorical variables and a Wilcoxon rank test for continuous variables and one-way ANOVA for group comparisons. Each ICP value was correlated with PI (mean of both sides), NPI (mean of both sides), and ONSD (mean of both sides). Correlation between continuous variables was evaluated using the Spearman correlation, with 95% confidence intervals, as appropriate. The Spearman index of correlation (*r*) was considered as “strong” (i.e., ≥ 0.7), “moderate” (i.e., 0.50–0.69), “weak” (0.25–0.50) or “poor” (i.e., < 0.25). The agreement between ICP and eICP was assessed using the Bland-Altman method; the mean bias and their limits of agreement (LoA) (as 2.2 times SD of the bias) were computed. The discriminative ability of NPI, ONSD, and PI to identify intracranial hypertension was evaluated using receiver operating characteristic (ROC) curves with the corresponding area under the curve (AUC) and related sensitivity and specificity. The estimated AUCs with their 95% CI for the combinations of the non-invasive tools were constructed using the logistic regression model with intracranial hypertension as the dependent variable and the combined non-invasive tools as the independent variables. Youden’s index was computed for each non-invasive method to identify the cut-off value with the best sensitivity and specificity to estimate intracranial hypertension. The comparison of each ROC curve was performed using the non-parametric DeLong method. A *p* < 0.05 was considered as statistically significant. Data were analyzed using IBM® SPSS® Statistics software, version 22 for Windows (IBM, Armonk, NY).

## Results

### Study population

A total of 195 patients underwent invasive ICP monitoring during the study period; 20 patients were younger than 18 years and 35 were excluded because ICP monitoring was inserted for ischemic stroke (*n* = 7), hydrocephalus (*n* = 6), or monitoring after at risk surgery for brain tumors (*n* = 11) or infection of ventriculo-peritoneal shunt (*n* = 11). Of 140 eligible patients, we excluded 40 patients (unstable ICP, *n* = 16; absent temporal window for TCD, *n* = 15; ocular trauma, *n* = 5; multiple sclerosis, *n* = 1; absence of arterial catheter, *n* = 3). Thus, 100 patients were included in the analysis, with TBI (*n* = 30), SAH (*n* = 47), or ICH (*n* = 23). The median Glasgow Coma Score on admission was 8 [5–12] and ICP assessment was performed 2 [2, 3] days after ICU admission. Other characteristics of the study cohort are shown in Table 1.

**Table 1:** Characteristics of the patients included in our cohort, according to underlying brain diseases

	ALL (***n*** = 100)	TBI (***n*** = 30)	SAH (***n*** = 47)	ICH (***n*** = 23)
**Demographics**				
**Age, years**	52 [44–62]	48 [31–62]	53 [46–59]	55 [44–65]^a^
**Male,*n*(%)**	55 (55)	20 (67)	22 (47)	11 (57)
**ICU length of stay (days)**	16 [11–23]	16 [12–23]	16 [12–23]	17 [11–23]
**GCS on admission**	8 [5–12]	7 [4–10]	9 [5–13]	9 [7–13]
**Days from admission to ICP assessment**	2 [2–3]	2 [2–3]	2 [2–3]	2 [2–3]
**Comorbidities**				
**Chronic heart failure,*n*(%)**	2 (2)	1 (3)	–	1 (4)
**Diabetes,*n*(%)**	8 (8)	–	4 (9)	4 (17)
**COPD/asthma,*n*(%)**	6 (6)	1 (3)	4 (9)	1 (4)
**Chronic hemodialysis,*n*(%)**	–	–	–	–
**Liver cirrhosis,*n*(%)**	4 (10)	2 (7)	2 (4)	–
**HIV,*n*(%)**	2 (2)	–	1 (2)	1 (4)
**Cancer,*n*(%)**	1 (1)	1 (3)	–	–
**Alcohol**	16 (16)	6 (20)	9 (19)	1 (4)
**On the day of ICP assessment**				
**Sedatives,*n*(%)**	39 (39)	17 (57)	19 (40)	3 (13)^a,b^
**Opioids,*n*(%)**	55 (55)	21 (70)	24 (51)	10 (43)
**Barbiturates,*n*(%)**	7 (7)	3 (10)	3 (6)	1 (4)
**Vasopressors,*n*(%)**	60 (60)	22 (73)	33 (70)	5 (22)^a,b^
**NMBAs,*n*(%)**	11 (11)	4 (13)	6 (13)	1 (4)
**Surgical procedures,*n*(%)**	43 (43)	19 (63)	13 (28)^a^	11 (48)
**Decompressive craniectomy,*n*(%)**	4 (4)	4 (13)	–	–
**Posterior fossa impairment,*n*(%)**	10 (10)	4 (13)	3 (6)	3 (13)
**GCS**	7 [3–10]	6 [3–8]	7 [3–11]	9 [3–12] ^a^
**Invasive and non-invasive ICP assessment**				
**Intracranial pressure, mmHg**	17 [12–25]	20 [13–26]	14 [12–23]	15 [12–23]
**Intracranial hypertension,*n*(%)**	37 (37)	15 (50)	15 (32)	7 (30)
**Cerebral perfusion pressure, mmHg**	77 [68–88]	75 [67–86]	78 [70–90]	82 [66–91]
**Mean ONSD, mm**	5.2 [4.8–5.8]	5.2 [4.8–5.7]	5.2 [4.9–5.9]	4.8 [4.5–5.6]
**Mean CBFDV, cm/s**	44 [34–56]	43 [38–52]	51 [36–60]	42 [32–50]
**Mean PI**	0.93 [0.83–1.16]	1.01 [0.90–1.15]	0.89 [0.81–1.14]	0.91 [0.83–1.11]
**eICP, mmHg**	18 [13–24]	19 [15–24]	16 [12–21]	18 [13–24]
**Mean NPI**	4.2 [3.8–4.6]	4.1 [3.6–4.3]	4.2 [3.6–4.6]	4.5 [4.0–4.7]
**Morbidity and outcomes**				
**MV during ICU stay,*n*(%)**	81 (81)	23 (77)	39 (83)	19 (83)
**Osmotic therapy during ICU stay,*n*(%)**	63 (63)	22 (73)	27 (57)	14 (61)
**Hypothermia during ICU stay,*n*(%)**	10 (10)	1 (3)	9 (19) ^a^	–
**ICU mortality,*n*(%)**	30 (30)	11 (37)	12 (26)	7 (30)
**GOS at hospital discharge**	3 [1–4]	4 [1–4]	4 [2–5]	3 [1–4]

*ICU* intensive care unit, *GCS* Glasgow Coma Scale, *COPD* chronic obstructive pulmonary disease, *HIV* human immunodeficiency virus, *NMBA* neuromuscular blocking agents, *ONSD* optic nerve sheath diameter, *CBFDV* cerebral blood flow diastolic velocity, *PI* pulsatility index, *ICP* intracranial pressure, *eICP* estimated intracranial pressure, *NPI* neurological pupil index, *MV* mechanical ventilation, *GOS* Glasgow Outcome Scale

^a^*p* < 0.05 vs. TBI

^b^*p* < 0.05 vs. SAH

### Invasive vs. non-invasive ICP measurement

**Fig. 1:**
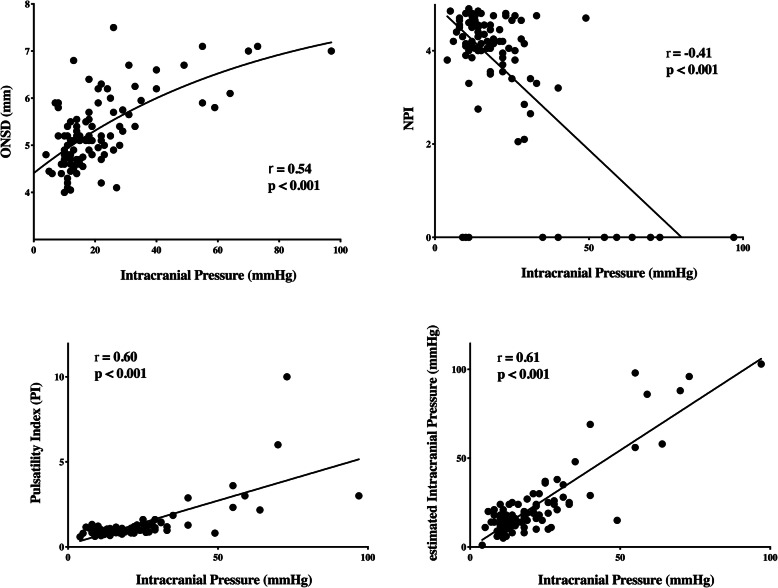
Scatterplot of invasive intracranial pressure (ICP, mmHg) and ICP estimates from the four non-invasive tool (optic nerve sheath diameter method, ONSD; neurological pupil index, NPI; pulsatility index, PI; estimated intracranial pressure, eICP)

**Fig. 2:**
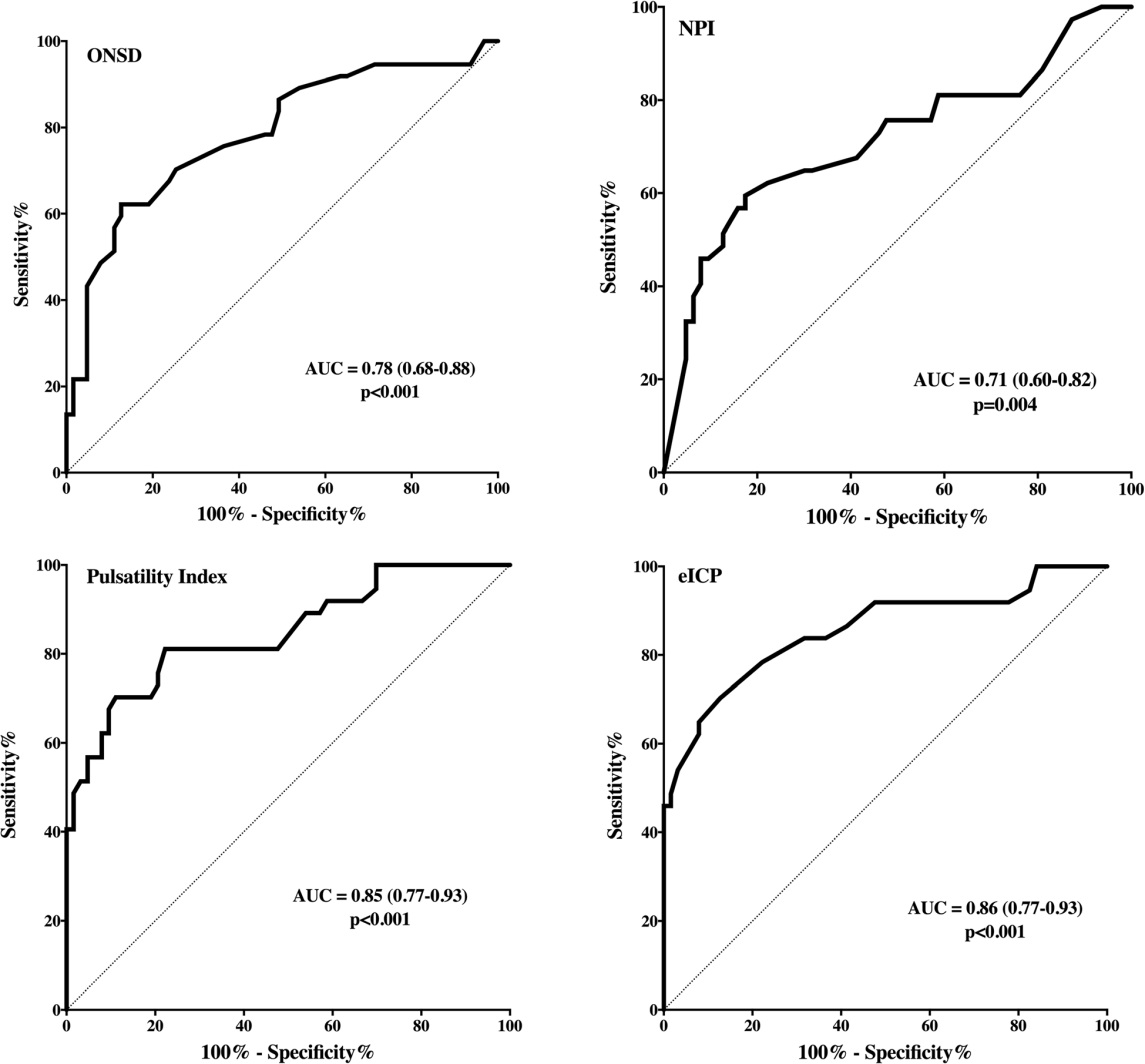
Receiver operating characteristic (ROC) analysis for different non-invasive ICP predictors (optic nerve sheath diameter method, ONSD; neurologic pupil index, NPI; pulsatility index, PI; estimated intracranial pressure, eICP) for estimate of intracranial hypertension (ICP > 20 mmHg). The area under the curve (AUC) is given with 95% confidence intervals

Median invasive ICP values were 17 [12–25] mmHg and intracranial hypertension was present in 37 patients. Median values from the different non-invasive techniques were ONSD 5.2 [4.8–5.8] mm, PI 1.1 [0.9–1.4], eICP 21 [14–29] mmHg, and NPI 4.2 [3.8–4.6]. The number of patients with abnormal ONSD, PI, eCIP, and NPI is reported in the Supplemental Material. There was a significant correlation between each technique and invasive ICP, although it was moderate for eICP (*r* = 0.61; *p* < 0.001), PI (*r* = 0.50; *p* < 0.001), and ONSD (*r* = 0.54; *p* < 0.001) and weak for NPI (*r* = − 0.41; *p* < 0.001—Fig. 1). The mean bias between ICP and eICP was − 1.63 mmHg (LoA − 20.70 to 17.44 mmHg—Supplemental Figure 1). The AUCs for estimating intracranial hypertension from the different devices are shown in Fig. 2; the AUCs for ONSD (*p* < 0.001), PI (*p* < 0.001), and eICP (*p* < 0.001) were significantly higher than that of NPI. ONSD > 5.3 mm had 70% sensitivity and 75% specificity to assess intracranial hypertension; PI > 0.97 had 81% sensitivity and 78% specificity; NPI < 4.1 had 65% sensitivity and 70% specificity; and an eICP > 20 mmHg had 73% sensitivity and 84% specificity. The AUCs for different combinations of the non-invasive tools to evaluate intracranial hypertension are shown in Table 2. The highest AUC (0.91 [0.84–0.97]) was obtained with the combination of ONSD with eICP and was similar to the AUC achieved with combination of all four indices.

**Table 2:** Area under the curve (AUC) for different combinations (using logistic regression model) of non-invasive tools for estimating intracranial hypertension

	ALL (***n*** = 100)	TBI (***n*** = 30)	SAH (***n*** = 47)	ICH (***n*** = 23)
**ONSD + PI**	0.89 [0.82–0.96]	0.88 [0.76–1.00]	0.92 [0.84–1.00]	0.84 [0.63–1.00]
**ONSD + eICP**	0.91 [0.84–0.97]	0.92 [0.81–1.00]	0.94 [0.87–1.00]	0.85 [0.62–1.00]
**ONSD + NPI**	0.80 [0.71–0.89]	0.80 [0.64–0.95]	0.80 [0.64–0.97]	0.90 [0.78–1.00]
**PI + eICP**	0.86 [0.77–0.94]	0.83 [0.69–0.98]	0.88 [0.76–1.00]	0.83 [0.61–1.00]
**PI + NPI**	0.85 [0.77–0.93]	0.80 [0.64–0.95]	0.86 [0.74–1.00]	0.88 [0.70–1.00]
**eICP NPI**	0.86 [0.78–0.94]	0.83 [0.69–0.98]	0.89 [0.77–1.00]	0.88 [0.73–1.00]
**ONSD + PI + eICP**	0.91 [0.84–0.97]	0.91 [0.80–1.00]	0.94 [0.88–1.00]	0.85 [0.64–1.00]
**ONSD + PI + NPI**	0.90 [0.83–0.96]	0.88 [0.77–1.00]	0.92 [0.84–1.00]	0.89 [0.74–1.00]
**ONSD + eICP + NPI**	0.91 [0.85–0.97]	0.92 [0.82–1.00]	0.94 [0.87–1.00]	0.89 [0.75–1.00]
**PI + eICP + NPI**	0.87 [0.79–0.94]	0.84 [0.69–0.98]	0.88 [0.76–1.00]	0.89 [0.74–1.00]
**ONSD + PI + eICP + NPI**	0.91 [0.85–0.97]	0.91 [0.80–1.00]	0.94 [0.88–1.00]	0.88 [0.73–1.00]

*ONSD* optic nerve sheath diameter, *PI* pulsatility index, *ICP* intracranial pressure, *eICP* estimated intracranial pressure, *NPI* neurological pupil index

### Invasive vs. non-invasive measurement in different forms of brain injury

The main differences among TBI, SAH, and ICH patients are shown in Table 1. For the TBI patients, the median Marshall score from the admission cerebral CT scan was 5 [3–6]. For the SAH patients, the median Fisher scale score from the admission cerebral CT scan was 4 [3–4]. Of the ICH patients, 12 (52%) had a supratentorial hemorrhage and in 13 (57%), the hemorrhage volume was estimated at > 30 mL.

There were no significant differences in ICP, ONSD, PI, eICP, or NPI among the three groups (Table 1). Correlations between invasive ICP and different tools for TBI, SAH, and ICH patients are presented in the Supplemental Material. The mean bias between ICP and eICP in the different forms of brain injury is shown in the Supplemental Figure 2. The AUCs for ONSD (*p* = 0.005), PI (*p* = 0.004), and eICP (*p* < 0.001) to estimate intracranial hypertension in TBI patients were significantly higher than the AUC for NPI. Similarly, the AUCs for ONSD (*p* = 0.001), PI (*p* < 0.001), and eICP (*p* < 0.001) to assess intracranial hypertension in SAH and ICH patients were significantly higher than the AUC for NPI.

### Correlation between non-invasive measurements

ONSD was significantly correlated with PI (*p* = 0.002), NPI (*p* = 0.02), and eICP (*p* < 0.001); however, the correlation was weak for all (PI, *r* = 0.30; NPI, *r* = − 0.22; eICP, *r* = 0.39). PI was significantly correlated with NPI (*p* = 0.006) and eICP (*p* < 0.001); however, the correlation was strong for eICP (*r* = 0.79) and moderate for NPI (*r* = − 0.27). eICP was significantly correlated with NPI (*p* = 0.003); the correlation was moderate (*r* = − 0.29).

## Discussion

In this study, including different forms of brain injury, the ICP estimated by the four non-invasive methods were significantly correlated with invasive ICP; however, the correlation was moderate to weak. The combination of ONSD and eICP had the best accuracy to estimate the occurrence intracranial hypertension. Similar results were observed when patients were analyzed according to the underlying brain pathology. To the best of our knowledge, this is the first study comparing these methods for non-invasive ICP estimation in the setting of multimodal neuromonitoring. For the first time, we present and compare the accuracy of 4 bedside tools not only in a large heterogeneous population, but also according to the type of brain pathology. Moreover, this is the first study exploring whether the combination of these non-invasive tools can reduce the possibility of error related to each single technique.

Knowledge of ICP is crucial when treating patients with acute brain injury, in particular after trauma [4, 22–24]. However, the indications for invasive ICP monitoring remain controversial in some brain conditions [4]. Although non-invasive ICP methods are not accurate enough to substitute for invasive ICP, non-invasive ICP estimation may be helpful and could be used as a “triage” method (e.g., identifying patients at high risk of developing intracranial hypertension who require specific monitoring and/or surveillance) or as a diagnostic tool in patients with unexplained alteration of consciousness outside the ICU [25–28]. As such, the non-invasive estimation of ICP has been widely investigated in brain-injured patients over the last few decades [29–34].

TCD has been widely used to assess ICP [31] and several TCD-derived indices have been proposed. Among those, results using PI have been conflicting. One study yielded significant correlations between ICP and PI [35]; however, other studies have demonstrated poor accuracy of this method to estimate ICP [10, 36]. Indeed, the elevation of PI has been misinterpreted as a precise indicator of increased cerebrovascular resistance, although it can also be influenced by PaCO_2_, pulse pressure, atherosclerosis, and sedation [36]. In our cohort, ICP estimated by PI was significantly correlated with invasive ICP, with acceptable sensitivity but low specificity to estimate intracranial hypertension.

Increasing values of ICP cause specific changes in the waveform analysis of TCD, with the diastolic flow velocity being primarily affected. Czosnyka et al. proposed a formula based on the diastolic flow velocity changes for non-invasive assessment of CPP (and therefore ICP), which showed an estimated error between estimated and measured CPP less than 10 mmHg in 71% of the examinations, and in 84% of the examinations, the error was less than 15 mmHg [11]. This formula has shown promising results in the estimation of ICP in both experimental and clinical settings [37–39]; however, a recent large prospective study [40] has challenged the utility of this method, reporting an AUC to assess intracranial hypertension of 0.35 with 0% sensitivity and 74% specificity. Several reasons could explain the differences in term of accuracy found by using eICP method across different studies [10, 33, 37, 40], such as the different designs of the studies (i.e., prospective vs. retrospective), the heterogeneity of the patients included and their cerebral pathology, the operator’s training and experience, or the hemodynamic and respiratory variables (in particular carbon dioxide) during the measurement. The ongoing multicenter international IMPRESSIT study (Invasive Versus Non-Invasive Measurement of Intracranial Pressure in Brain Injury Trial; ClinicalTrials.gov NCT02322970) will provide additional data to solve this conundrum. Our results suggest a relevant role for such an approach to assess ICP; among the studied non-invasive indices, eICP had the highest correlation and accuracy with invasive ICP in the total population and in the different subpopulations of brain injury.

The ultrasonographic measurement of ONSD is gaining popularity for non-invasive ICP estimation and has been investigated in different clinical scenarios showing a good correlation with invasive ICP values and good inter- and intra-observer variability [41, 42]. The optic nerve is surrounded by a subarachnoid space and, in the intra-orbital part of the subarachnoid space, is distensible and can therefore swell if cerebrospinal fluid pressure increases. In a prospective observational study on patients with brain injury, ONSD, when compared with venous TCD, PI, and eICP, showed the highest accuracy to estimate ICP [10]. Similarly, a recent meta-analysis [42] showed an AUC of 0.94 for ONSD to evaluate intracranial hypertension, although different cut-offs were identified across studies and most of the cohorts included only TBI patients. Our results are in agreement with these findings; in our cohort, ONSD had a good correlation with ICP and good sensitivity and specificity regardless of the type of brain injury.

Recently, NPI has been suggested as a valuable technique for early recognition of increased ICP [17]. The automated pupillometer enables a quantitative and non-operator-dependent evaluation of pupillary function [18]. The parasympathetic oculomotor nuclei in the midbrain are particularly sensitive to brainstem compression and altered pupillary function can indicate an expanding supratentorial mass with subsequent increases in ICP. Chen et al. [17] demonstrated that a relationship exists between NPI values < 3 and increased ICP, with abnormal NPI values present before the increase in ICP. A recent study including 54 TBI patients [43] demonstrated that sustained episodes of elevated ICP were associated with a concomitant decrease in the NPI, which increased with the decrease in ICP after osmotic therapy. However, in our study, NPI had the lowest accuracy for estimating of intracranial hypertension and its addition to the other indices did not significantly improve their accuracy.

Taken together, important disagreement exists in the literature on the role of non-invasive ICP methods. The observed conflicting results may be related to several factors including the heterogeneity of brain injuries included in the different studies, different methodologies, different expertise of the operators, and inter-observer variability. In our cohort, results from subgroups of different types of brain injury suggest that each method has different accuracy depending on the type of brain injury. For example, we found that PI had good correlation with ICP in TBI and ICH and a weak correlation in SAH; NPI had a good correlation with ICP in SAH, but a weak correlation in TBI; by contrast, ONSD and eICP had good accuracy in all three types of brain injury. The discrepancies across different types of brain injury may be related to the pathophysiological effect of ICP on brain function in the different conditions, and potentially to different critical ICP thresholds to worsen brain damage in different conditions. In particular, as each of the non-invasive methods relies on different pathophysiological mechanisms underlying intracranial hypertension (i.e., increased cerebrospinal fluid pressure for ONSD, pupillary changes for NPI, alteration of flow waveform for TCD), it is plausible that only their combination would improve the estimation of altered brain compliance and increased ICP. This hypothesis is in agreement with a recent study [10] comparing four different non-invasive ICP methods (ONSD, PI, eICP, and venous TCD) which demonstrated that the combination of ONSD and venous TCD was significantly more accurate than use of a single method.

This study has several limitations. First, the number of patients included was relatively small for each subgroup of patients, and only one measurement per patient was obtained; more measurements for each patient over time would have strengthened our findings and permitted to assess also estimation of ICP changes. Moreover, some confounding factors, such as the effects of PaCO_2_, imaging, body temperature, or cumulative doses of sedatives, were difficult to assess, but can potentially have an important effect, especially on TCD-derived measurements. Also, timing from ICU admission to assessment was variable between patients. Third, the vast majority of measurements were obtained in patients with relatively well-controlled ICP, and we cannot extrapolate these findings with highly variable ICP values. Fourth, all measurements were performed by the same operator, which can increase the reliability of the observations, but also prevents assessment of interobserver variability. Fifth, averaging measurement from both sides, as for ONSD, could be questionable and one may argue that ONSD of a specific side (i.e., the injured side or the same side or where ICP probe was placed) would be more adequate. However, most studies also used average data on both cerebral sides and comparison of the two approaches (i.e., injured side vs. averaged values) have not been adequately addressed in other reports.

## Conclusions

In this study, non-invasive methods to estimate ICP based on TCD, pupillometry, or optic nerve ultrasonography were significantly correlated with invasive ICP values, although the correlation was moderate to weak. The best accuracy was found for eICP method. The multimodal combination of such indices may increase the ability to identify intracranial hypertension.

## Supplementary information

**Additional file 1 Supplemental Table 1.** Number of abnormal values for each monitoring tool, according to the different forms of brain injury. **Supplemental Figure 1.** Correlations and Bland-Altman graphs for ICP and estimated ICP (eCIP). The continuous line shows the mean difference (bias) and the dotted lines show the limits of agreement (2.2 ± SD of the bias). **Supplemental Figure 2.** Correlations and Bland-Altman graphs for ICP and estimated ICP (eCIP). The continuous line shows the mean difference (bias) and the dotted lines show the limits of agreement (2.2 ± SD of the bias). The mean bias between ICP and eICP were: − 1.37 mmHg (LoA − 21.78 to 19.05 mmHg) for TBI; − 0.68 mmHg (LoA − 19.67 to 18.30 mmHg) for SAH; − 3.91 mmHg (LoA − 21.36 to 13.54 mmHg) for ICH.

## Data Availability

Yes
