# Supplementary material for: Multimodal non-invasive assessment of intracranial hypertension: an observational study
Source: Crit Care. 2020 Jun 26;24:379. doi: 10.1186/s13054-020-03105-z (PMC7318399; doi:10.1186/s13054-020-03105-z)
Supplement: Supplementary file 1 — Additional file 1 Supplemental Table 1. Number of abnormal values for each monitoring tool, according to the different forms of brain injury. Supplemental Figure 1. Correlations and Bland-Altman graphs for ICP and estimated ICP (eCIP). The continuous line shows the mean difference (bias) and the dotted lines show the limits of agreement (2.2 ± SD of the bias). Supplemental Figure 2. Correlations and Bland-Altman graphs for ICP and estimated ICP (eCIP). The continuous line shows the mean difference (bias) and the dotted lines show the limits of agreement (2.2 ± SD of the bias). The mean bias between ICP and eICP were: − 1.37 mmHg (LoA − 21.78 to 19.05 mmHg) for TBI; − 0.68 mmHg (LoA − 19.67 to 18.30 mmHg) for SAH; − 3.91 mmHg (LoA − 21.36 to 13.54 mmHg) for ICH. [file 13054_2020_3105_MOESM1_ESM.docx]

**Supplemental Material**

**Multimodal non-invasive estimation of intracranial hypertension: an observational study**

*Chiara ROBBA,^1,2^ Selene POZZEBON,^3^ Bedrana MORO, ^3^ Jean-Louis VINCENT, ^3^ Jacques CRETEUR, ^3^ Fabio Silvio TACCONE^3^*

^1^Policlinico San Martino, IRCCS For Oncology and Neuroscience, Department of Integrated Surgical and Diagnostic Science, University of Genova, Italy

^2^Neurosciences Critical Care Unit, Addenbrooke’s Hospital, University of Cambridge, United Kingdom.

^3^Department of Intensive Care Medicine, Erasme Hospital, Université Libre de Bruxelles, Brussels, Belgium

*Abnormal values for each monitoring tool*

The number of patients with abnormal NPI, PI, ONSD and eICP values was 17 (17%), 21 (21%), 20 (20%) and 35 (35%), respectively (p=0.01). The differences in the number of patients with abnormal NPI, PI, ONSD and eCIP between the three forms of brain injury are shown in Supplemental Table 1.

*Invasive vs. Non-invasive measurement in different forms of brain injury*

In the TBI subgroup, there was a significant moderate correlation between invasive ICP and ONSD (r =0.53, p=0.002), PI (r =0.60, p<0.001) and eICP (r= 0.66, p<0.001), but no correlation with NPI (r =-0.16, p=0.37). The AUC to predict intracranial hypertension was 0.78 [95% CIs 0.62-0.95] for ONSD, 0.79 [95% CI: 0.63-0.96] for PI, 0.83[95% CI: 0.69-0.98] for eICP and 0.61 [95% CI: 0.49-0.83] for NPI. In particular, a ONSD > 5.3 mm had 67% sensitivity and 73% specificity to predict intracranial hypertension; a PI > 1.10 had 61% sensitivity and 80% specificity to predict intracranial hypertension; a NPI < 4.0 had 61% sensitivity and 73% specificity to predict intracranial hypertension; an eICP > 20 mmHg had 67% sensitivity and 87% specificity to predict intracranial hypertension.

In the SAH subgroup, there was a significant correlation between invasive ICP and each technique, although it was strong for NPI (r=-0.77, p<0.001) and eICP (r=0.88, p<0.001) and moderate for ONSD (r =0.61, p<0.001) and PI (r = 0.53, p<0.001). The AUC to predict intracranial hypertension was 0.78 [95%CIs 0.62-0.94] for ONSD, 0.79 [95%CIs 0.63-0.96] for PI, 0.68 [95%CIs 0.61-0.75] for eICP and 0.70 [95%CIs 0.51-0.89] for NPI. In particular, a ONSD > 5.7 mm had 73% sensitivity and 85% specificity to predict intracranial hypertension; a PI > 0.98 had 71% sensitivity and 100% specificity to predict intracranial hypertension; a NPI < 4.0 had 60% sensitivity and 71% specificity to predict intracranial hypertension; an eICP > 20 mmHg had 80% sensitivity and 90% specificity to predict intracranial hypertension.

In the ICH group, there was a significant moderate correlation between invasive ICP and each technique; ONSD (r =0.56, p<0.001), PI (r =0.68, p<0.001), eICP (r =0.53, p<0.001) and NPI (r = -0.57, p<0.001). The AUC to predict intracranial hypertension was 0.90 [95%CIs 0.77-0.99] for ONSD, 0.69 [95%CIs 0.65-0.78] for PI, 0.97 [95%CIs 0.96-0.98] for eICP and 0.73 [95%CIs 0.64-0.87] for NPI. In particular, a ONSD > 5.7 mm had 72% sensitivity and 94% specificity to predict intracranial hypertension; a PI > 1.23 had 71% sensitivity and 100% specificity to predict intracranial hypertension; a NPI < 4.0 had 71% sensitivity and 94% specificity to predict intracranial hypertension; an eICP > 24 mmHg had 71% sensitivity and 94% specificity to predict intracranial hypertension.

**Supplemental Table 1.** Number of abnormal values for each monitoring tool, according to the different forms of brain injury.

|  | **ALL**  **(n=100)** | **TBI**  **(n=30)** | **SAH**  **(n=47)** | **ICH**  **(n=23)** |
| --- | --- | --- | --- | --- |
| **Abnormal NPI, n** | 17 | 4 | 8 | 5 |
| **Abnormal PI, n** | 21 | 7 | 9 | 5 |
| **Abnormal ONSD, n** | 20 | 4 | 11 | 5 |
| **Abnormal eICP, n** | 35 | 12 | 14 | 9 |
| **Intracranial Hypertension, n** | 37 | 15 | 15 | 7 |

ONSD = optic nerve sheath diameter; PI = pulsatility index; ICP = intracranial pressure; eICP = estimated intracranial pressure; NPI = neurological pupil index; TBI = traumatic brain injury; SAH = subarachnoid hemorrhage; ICH = intracranial hemorrhage

**Supplemental Figure 1.** Correlations and Bland-Altman graphs for ICP and estimated ICP (eCIP). The continuous line shows the mean difference (bias) and the dotted lines show the limits of agreement (2.2±SD of the bias).


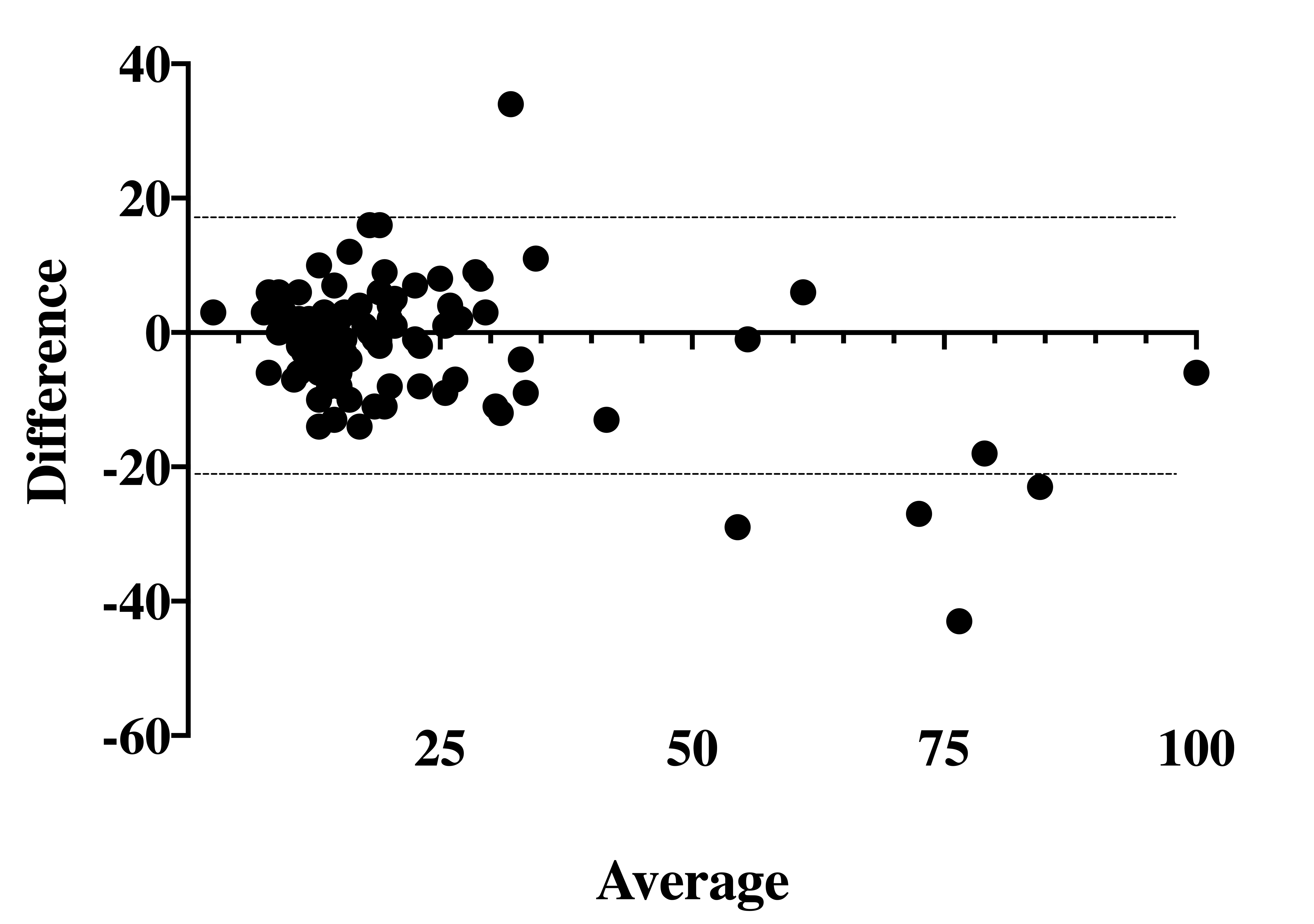


**Supplemental Figure 2.** Correlations and Bland-Altman graphs for ICP and estimated ICP (eCIP). The continuous line shows the mean difference (bias) and the dotted lines show the limits of agreement (2.2±SD of the bias). The mean bias between ICP and eICP were: -1.37 mmHg (LoA -21.78 to 19.05 mmHg) for TBI; -0.68 mmHg (LoA -19.67 to 18.30 mmHg) for SAH; -3.91 mmHg (LoA -21.36 to 13.54 mmHg) for ICH.


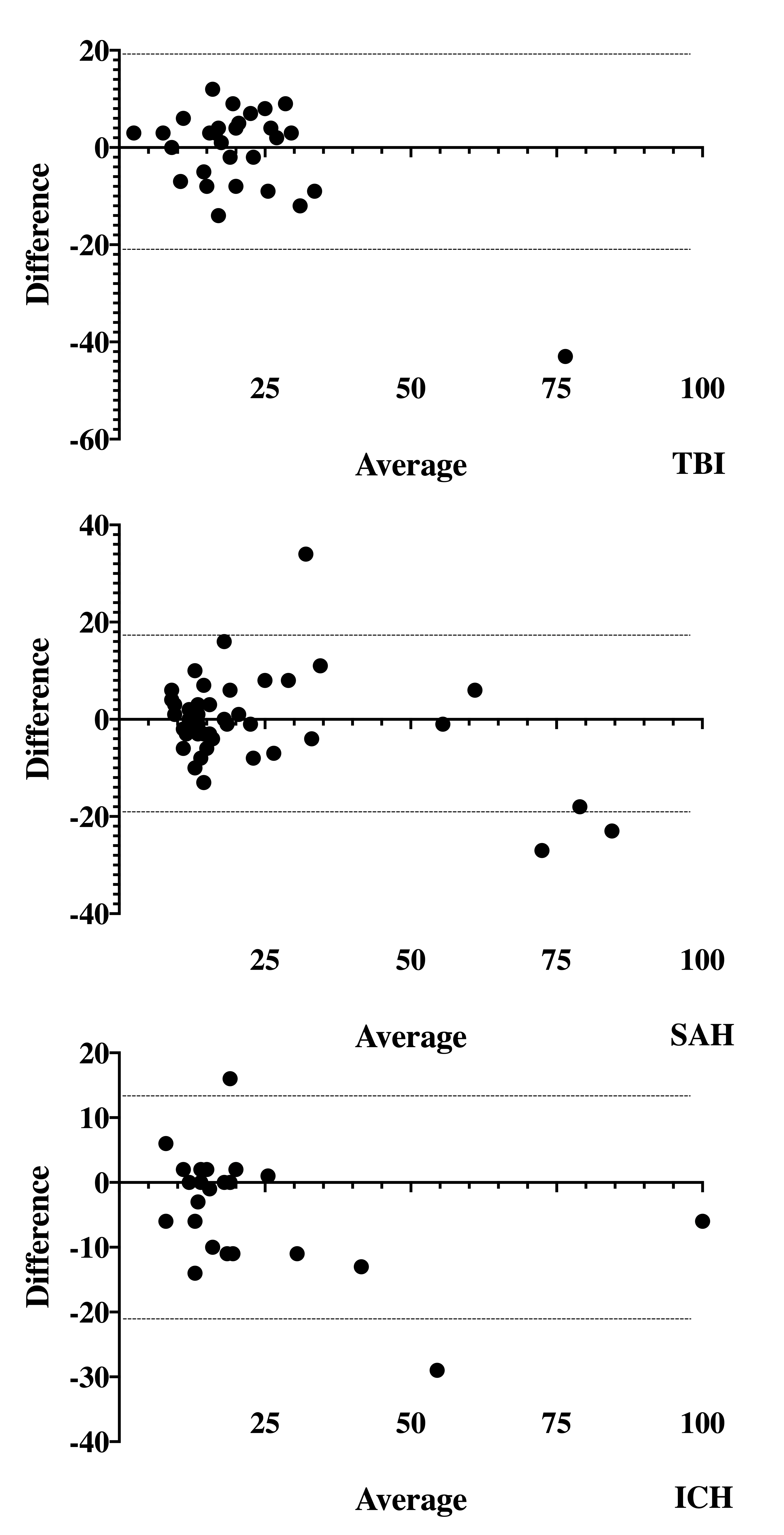


TBI = Traumatic Brain Injury; SAH = Subarachnoid Hemorrhage; ICH = Intracranial Hemorrhage
